# Adsorption characteristics and mechanism of ammonia nitrogen and phosphate from biogas slurry by Ca^2+^-modified soybean straw biochar

**DOI:** 10.1371/journal.pone.0290714

**Published:** 2023-08-25

**Authors:** Xiaomei Wu, Meifeng Ye, Jinglong Wang, Feilong Wu, Cenwei Liu, Zhangting Li, Daiyan Lin, Rilong Yang

**Affiliations:** 1 Agricultural Engineering Institute, Fujian Academy of Agricultural Sciences, Fuzhou, Fujian, China; 2 College of Material Engineering, Fujian Agriculture and Forestry University, Fuzhou, China; 3 Key Laboratory of Algal Biology, Institute of Hydrobiology, Chinese Academy of Sciences, Wuhan, China; 4 Institute of Agricultural Ecology, Fujian Academy of Agricultural Sciences, Fuzhou, Fujian, China; Universiti Teknologi Petronas: Universiti Teknologi PETRONAS, MALAYSIA

## Abstract

The utilization of biogas slurry is critical for the sustainable development of animal husbandry. Biomass carbon adsorption is a feasible method for the recycling of nutrients from biogas slurry. However, research on the co-adsorption of ammonia nitrogen and phosphate is scarce. Herein, soybean straw was utilized as the raw material to prepare Ca^2+^-modified biochar (CaSSB), which was investigated for its ammonia nitrogen and phosphate adsorption mechanisms. Compared with natural biochar (SSB), CaSSB possesses a high H/C ratio, larger surface area, high porosity and various functional groups. Ca^2+^-modified soybean straw biochar exhibited excellent adsorption performance for NH_4_^+^–N (103.18 mg/g) and PO_4_^3−^−P (9.75 mg/g) at pH = 6, using an adsorbent dosage of 2 g/L. The experimental adsorption data of ammonia nitrogen by CaSSB corresponded to pseudo-second-order kinetics and the Langmuir isotherm model, suggesting that the adsorption process was homogeneous and that electrostatic attraction might be the primary adsorption mechanism. Meanwhile, the adsorption of phosphate conformed to pseudo-second-order kinetics and the Langmuir–Freundlich model, whose mechanism might be attributed to ligand exchange and chemical precipitation. These results reveal the potential of CaSSBs as a cost-effective, efficient adsorbent for the recovery of ammonium and phosphate from biogas slurry.

## Introduction

Livestock and poultry manure production has increased as a result of advancements in intensive and large-scale farming. Anaerobic digestion has emerged as a popular technology for treating these manures, offering advantages such as low cost, high efficiency and ease of operation. In China, approximately 90% of farm manure is treated using biogas anaerobic digestion [1]. Nevertheless, the proliferation of biogas plants has led to an increase in the production of biogas slurry, posing a challenge for its harmless resource treatment [2]. Biogas slurry, a by-product of anaerobic fermentation, contains various biologically active substances, including nitrogen, phosphorus, potassium, amino acids, trace elements, organic acids and humic acids [3]. Discharging untreated biogas slurry into water causes environmental pollution and wastes valuable nitrogen and phosphorus resources [4]. Various treatment technologies have been previously utilised, including coagulation–flocculation, catalytic oxidation and A^2^/O. However, their widespread application has been hindered by several limitations, including high cost, unstable operation and inadequate phosphorus treatment [5–8]. In recent years, extensive attention has been paid to the recovery of nitrogen and phosphorus nutrients from biogas slurry, driven by policies promoting the resource utilisation of livestock and poultry manure.

There are several frequently-used technologies for recovering nitrogen and phosphorus from biogas slurry recovery, including crystallisation, ultrafiltration, filtration, reverse osmosis, phytoremediation, and so on [9, 10]. These methods offer a twofold advantage by facilitating nutrient recovery and generating clean water from biogas slurry. The resulting clean water can be either directly discharged into water bodies or used for non-domestic applications. Nevertheless, the high expenses and substantial chemical consumption pose obstacles to the extensive implementation of these technologies. Adsorption technology is a straightforward, effective and economical technique for nutrient recovery from biogas slurry. It reduces nitrogen and phosphorus efflux pollution, prevents resource waste and enables the reuse of the slurry as a soil conditioner [11]. However, commonly employed adsorbents, such as commercial activated carbon [12], minerals [13] and zeolites [14], are usually expensive and are limited in their potential applicability, characterised by low utilization value. The biological nutrient removal efficiency is considerably influenced by the choice of an appropriate adsorbent in this approach [15]. Therefore, it is highly desirable to develop a new type of adsorbent with low production cost and effective adsorption properties.

Biochar is a porous carbon-rich by-product generated via gasification or pyrolysis of agricultural and forestry biomass under anaerobic conditions [16]. Biochar has been widely adopted in the fields of water purification and soil amelioration owing to its high porosity, stable aromatic structure, excellent stability and rich functional groups [17–19]. The physicochemical properties of biochar are highly dependent on the biomass source and various carbonization processes. Thus, biochars derived from different raw materials exhibit varying adsorption capacities for nitrogen and phosphorus [20]. To achieve simultaneous adsorption, biochars have been modified with metal cations such as iron [21], calcium [22], aluminium [23] and magnesium [24], which remarkably enhance their pollutant adsorption capacity. Among them, calcium is a versatile resource known for its low cost and ease of operation, and it exhibits remarkable binding capabilities for phosphorus [25]. Furthermore, Ca^2+^ is a medium element required for plant growth. Liu [26] impregnated ramie stem biochar with calcium chloride, determining that biochar loaded with calcium facilitated the precipitation reaction between calcium and phosphorus, chemisorption being the primary mechanism behind this process. Antunes [27] reported that the phosphate adsorbed on Ca^2+^-doped biochar could be converted into hydroxyapatite crystals. Zhang et al. [20] found that the removal of ammonia nitrogen occurs mainly via ion exchange. Ca^2+^-modified biochar not only exhibits excellent adsorption capacity for nitrogen and phosphorus, but also exhibits environmental benefits [28, 29].

Selecting abundant and easily available biomass raw materials promotes the application of biochar. Soybean straw, the agricultural waste obtained after soybean harvest, is cost effective and easily available. Using soybean straw to produce biochar can reduce the production cost and decrease environmental pollution. Several studies reported the removal of heavy metals, dyes and organic matter using straw charcoal. However, studies reporting on the joint adsorption of ammonia nitrogen and phosphate in biogas slurry by calcium-impregnated biochar are scarce. Moreover, the specific mechanism of ammonium and phosphate removal through Ca^2+^-modified biochar derived from soybean straw remains unclear.

This study investigates the performance and mechanism of Ca^2+^-modified soybean straw–based biochar (CaSSB) in adsorbing and recovering ammonium and phosphorus from biogas slurry. The nitrogen and phosphorus pollution within the biogas slurry is primarily derived from ammonium and phosphate that facilitates the utilization of ammonia nitrogen and phosphate as inspection indicators [30]. The objectives of this study can be summarised as follows: (1) preparation of calcium chloride impregnated biochar; (2) characterisation of the physicochemical properties of Ca^2+^-modified soybean straw biochar via X-ray diffraction (XRD), scanning electron microscopy (SEM), Fourier transform infrared spectroscopy (FTIR), Brunauer Emmett Teller (BET) and zero energy thermonuclear assembly analysis (zeta); (3) evaluating the effects of dosage, pH, coexisting ions, adsorption kinetic and adsorption isotherms on adsorption and recovery; (4) investigating the CaSSB adsorption mechanism of ammonium and phosphate. This study proposes a promising method to prepare an efficient absorbent based on agricultural waste for ammonia nitrogen and phosphate recovery from biogas slurry.

## Materials and methods

### Soybean straw (SS) and biogas slurry

Soybean straw was obtained from a farm in the suburb of Fuzhou City, Fujian Province, China. The biomass was chopped, soaked in deionized water and placed in an oven for drying at 105°C for 12 h until constant weight was achieved. The dried soybean straw was pulverized and sieved through a 30-mesh sieve (particle size of 0.6 mm). After grinding, the powder was dried at 105°C for 24 h in an oven and cooled in the desiccator before carbonization and impregnation. Results of the elemental and proximate analyses of the raw soybean straw and biochars are listed in Table 2. Biogas slurry was obtained from large-scale pig farms, Xingyuan agriculture and animal husbandry technology Corporation, located in in the suburb of Fuzhou City, Fujian Province, China. The biogas slurry was refrigerated prior to the experiment. Table 1 lists the chemical characteristics of the biogas slurry.

**Table 1:** Characteristics of biogas slurry (n = 3, SD).

Item	unit	Mean-value	Item	unit	Mean-value
pH	/	8.35 ± 0.32	Cu	mg·kg^−1^	2.54 ± 0.41
COD_cr_	mg·L^−1^	1173.41 ± 10.32	Zn		5.07 ± 0.22
NH_4_^+^–N		474.54 ± 7.89	Ni		0.04 ± 0.01
PO_4_^3—^P		50.91 ± 1.70	As		0.11 ± 0.04
DO		3.11 ± 0.21	Cd		0.02 ± 0.01
BOD_5_		724.64 ± 8.46	Cr		0.16 ± 0.02
NO^3—^N		3.16 ± 0.17	Pb		0.01 ± 0.01

### Chemical reagents

The chemical reagents involved in this work, including KH_2_PO_4_, NH_4_Cl, NaOH, HCl, CaCl_2_, CuCl_2_, ZnCl_2_, KNO_3_, K_2_SO_4_, were all of analytical grade and purchased from the Shanghai Aladdin Chemical Reagent Company.

### Preparation of biochars

Carbonization: A slow pyrolysis process was selected to increase the biochar yield. Soybean straw was processed in a vacuum tube furnace (DSK-60A, Dongguan, China) at a temperature of 350°C for 2 h under nitrogen atmosphere, and then cooled to room temperature under nitrogen protection. The heating rate was 10°C/min, and the nitrogen flow rate was 150 mL/min during the preparation of biochar. The biochar was designated as SSB.

Modification: The preparation of Ca^2+^-modified SSB (CaSSB) comprised two steps. First, the biochar was acid-washed with 6 mol/L hydrochloric acid for 1 h, and the solid–liquid ratio was 1:15 (m:v). Subsequently, the biochar was filtered and rinsed with deionized water five times to remove residual acid, and dried at 75°C for 12 h. Second, 100 mL of 1.5 mol/L CaCl_2_ solution was added to 2.00 g of SSB to achieve a weight ratio of calcium to SSB (M(Ca)/M(SSB)) of 3.0. Then, the mixed solution was reacted in a magnetic stirrer for 24 h. Finally, the solid matter was dried at 75°C after removing the filtrate to obtain Ca^2+^-modified SSB (CaSSB).

### Physicochemical characterisation

The organic composition in the biomass and biochar was analysed by the elemental analyser (LECO ONH2000, Elementar, USA). In a helium atmosphere, O was analysed at 1250°C; C, H and N were analysed at 1050°C and sulphur was analysed at 1350°C in an oxygen environment. The moisture and ash content were determined based on a dry basis. BET (APSP 2460, micromeritics, USA) was used to measure the specific surface area and total pore volume of the biochar, and BET and BJH model methods were employed for calculation and analysis. The zeta potential of SS, SSB and CaSSB were gauged using the Malvern Zetasizer Nano ZS90 (Malvern Instruments, UK, 2012). The surface morphology of the biochar was observed and characterized by SEM (TESCAN MIRA4). XRD (Panalytical X’Pert’3 Powder, Netherlands) was used to identify the crystallinities in the biochar after Ca^2+^ loading. FTIR (Thermo Fisher Scientific, Nicolet iS20, USA) was employed for infrared characterization of the biochar. The samples were qualitatively scanned by the potassium bromide solid tablet method under the conditions of scanning wave number 4000–400 cm^−1^ and resolution of 1 cm^−1^.

### Adsorption experiment

Batch experiments were conducted to obtain adsorption data dependence on the pH and dose of CaSSB. The pH of ammonium phosphate solution was controlled with diluted HCl or NaOH, and the pH level was maintained throughout the entire absorption experiment. To select the optimal adsorption dosage, the adsorption capacity of CaSSB for ammonia and phosphate was tested using adsorption dosages of 0.05, 0.10, 0.15, 0.20, 0.30, 0.40 and 0.50 g at a pH of 6. To investigate the influence of pH on ammonia nitrogen and phosphate, the initial pH was adjusted to 3, 4, 5, 6, 7, 8, 9 and 10 by HCl (0.1 M) and NaOH (0.1 M) at the CaSSB dosage of 2 g/L. The presence of various ions in the biogas slurry requires us to take into account the influence of common coexisting ions on the adsorption process. The various concentrations of K^+^, Ca^2+^, Cu^2+^, Zn^2+^, Cl^−^, CO_3_^2−^, SO_4_^2−^ and NO_3_^−^ were respectively added into the 474.00 mg/L ammonium and 50.00 mg/L phosphate solution before the addition of 2 g/L CaSSB. To assess the applicability of the CaSSB adsorbent for actual wastewater, 0.1 g CaSSB was added to the 50 mL biogas slurry. A 250-mL glass Erlenmeyer flask test container and test adsorption solutions, each with a volume of 50 mL, was used. The adsorption process was carried out at room temperature with constant shaking at a speed of 150 r/min for 24 h. When the adsorption was complete, the sample to be tested was filtered by a 0.45-μm filter to assess ammonia nitrogen and phosphate quantities. All experiments in this study were conducted in triplicate to reduce errors. Eq (1) calculates the removal rate of ammonia nitrogen and phosphate by samples:

$$x=\frac{\left(S_{0}-S_{e}\right)}{S_{0}}\times 100\%$$ (1)

Among them, *x* represents the removal rate (%), and *S*_*0*_ and *S*_*e*_ represent the initial and equilibrium state concentrations of ammonia nitrogen and phosphate (mg/L), respectively.

The adsorption capacity of ammonia and phosphate can be calculated according to Eq (2):

$$q=\frac{(S_{0}-S_{e})\times V}{1000\times m},$$ (2)

where *V* represents the volume of reaction solution (mL) and *m* represents the weight of added biochar (g).

Nessler’s reagent was used to determine the concentration of ammonia nitrogen, and the phosphate concentration was determined by antimony–Mo blue.

### Adsorption kinetic analysis

For the kinetic adsorption experiments, NH_4_Cl and KH_2_PO_4_ were dissolved in deionized water to prepare the simulated sewage of ammonium and phosphate. The ammonia nitrogen concentration in the solution was 30 mg/L, whereas the phosphate concentration was 20 mg/L. To explore the adsorption kinetics of ammonia nitrogen and phosphate, batch experiments were conducted in triplicate. A total of 0.4 g of the adsorbent was introduced to 200 mL of ammonium phosphate solution. Prior to conducting the experiments, the pH_initial_ of the sorption solution was set at 6. The mechanical shaker was set at 150 r/min and the vessels were left to shake at room temperature. The collected suspension was filtered at specific times (ranging from 5 to 1440 min). To determine the ammonia nitrogen and phosphate adsorption kinetics of the CaSSB, three classical models the pseudo-first-order kinetics equation, pseudo-second-order kinetics and the intraparticle diffusion model were employed [31].


$$q_{t}=q_{e}(1-e^{-k_{1}t})$$ (3)

$$\frac{t}{q_{t}}=\frac{1}{k_{2}q_{e}{}^{2}}+\frac{t}{q_{e}}$$ (4)

$$q_{t}=k_{3}t^{1/2}+a$$ (5)

Herein, *q*_*e*_ (mg/g) and *q*_*t*_ (mg/g) respectively denote the quantity of ammonia nitrogen or phosphate adsorbed at equilibrium at a specific time t. *k*_*1*_ (min^−1^) and *k*_*2*_ (g/(mg·min)) represent the reaction rate constants of the pseudo-first-order model and pseudo-second-order model, which are calculated from the plot of ln(*q*_*e*_-*q*_*t*_) versus *t* and *t*/*q*_*t*_ versus *t*, respectively. *k*_*3*_ (mmol kg^−1^ h^−0.5^) denotes the intra-particle diffusion rate constant, and *a* (nmol/kg) is a constant.

### Adsorption isotherm analysis

Herein, CaSSB (2 g/L) was mixed with seven groups of ammonium phosphate solutions, each with concentrations ranging from 0 to 100 mg/L. The mixture was placed in a shaking incubator at 25°C and 150 r/min. All samples were equilibrated for 24 h, and the concentrations of ammonia nitrogen and phosphate at equilibrium were measured. The Langmuir, Freundlich and Langmuir–Freundlich isotherm models were used for fitting the test data [32]. The Langmuir model, assuming uniform monolayer surface adsorption, can be expressed by Eq (6):

$$\frac{s_{e}}{q_{e}}=\frac{1}{q_{max}K_{L}}+\frac{s_{e}}{q_{max}}$$ (6)

The Freundlich model assumes that the adsorption behaviour on the adsorbent surface is heterogeneous, and its expression is given by Eq (7):

$$q_{e}=K_{F}S_{e}{}^{\frac{1}{n}}$$ (7)

The Langmuir–Freundlich is expressed as Eq (8):

$$q_{e}=\frac{q_{\max}K_{LF}S_{e}{}^{n}}{1+K_{LF}S_{e}{}^{n}}$$ (8)

In this equation, *q*_*max*_ (mg/g) represents the maximum adsorption capacity; *K*_*L*_ is the affinity constant; *K*_*F*_, *K*_*LF*_ and 1/*n* represent the constants.

### Data analysis

Excel 2018 was used to summarize and organize the data, and Origins 8.0 was used to perform regression analysis and plot the data on the adsorption of ammonia nitrogen and phosphate by biochar. Error bars represent the standard deviation (SD), calculated from the experiments performed in triplicate.

## Results and discussion

### Characterisation of biochar

#### Elemental composition and Zeta potential of biochars

The elemental composition of the pristine material and biochars used in this experiment are presented in Table 2. The results showed that charcoal was the major component of the biochars, indicating that the adsorbent had carbonaceous properties. After carbonization and modification of the soybean straw, the proportion of carbon and nitrogen content increased, while the proportion of oxygen and hydrogen elements decreased. Among them, the contents of oxygen and hydrogen in CaSSB were higher than those in SSB, whereas the contents of carbon and nitrogen exhibited the opposite trend. In addition, the ash content of CaSSB was lower than that of SSB, and the calcium content was 8.65 times as much as that of SSB, indicating that calcium was loaded on soybean straw carbon. The H/C and O/C ratios remained widely used as indicators for describing the degree of carbonization and evaluating the aromaticity and stability of biochar [33]. Compared with SSB, the H/C and O/C ratio of CaSSB increased by 0.23 and 0.21, respectively. Based on these results, CaSSB is likely to possess higher hydrophilicity than SSB, as it exhibits a higher O/C ratio (0.44 vs 0.23).

**Table 2:** Elemental analysis and proximate analysis (wt%) of raw SS and biochars.

sample	Elemental /%				H/C	O/C	Ash	Ca	Zeta potential
	C	H	N	O			/%	/%	/mv
SS	39.43	6.45	1.42	39.47	1.96	0.75	4.31	1.06	−19.8
SSB	72.83	3.64	1.64	22.29	0.60	0.23	10.19	1.10	−23.4
CaSSB	58.53	4.03	1.78	34.66	0.83	0.44	7.41	9.52	−29.6

The zeta potential can be used to represent the strength of the inter-particle interaction force inside the samples [34]. As the absolute value of the zeta potential increases, the dispersed particles reduce in size and the entire system increases in stability. The zeta potentials of SS, SSB and CaSSB measured at a pH of 7 are shown in Table 2. The three materials were all negatively charged. Yao et al. [35] suggested that the increase in the zeta potential of carbon materials led to superior adsorption performance. From the absolute value of the zeta potential, the value of CaSSB was greater than that of SS and SSB, indicating that CaSSB had better dispersion and might exhibit excellent adsorption performance for ammonium.

#### SEM and XRD

**Fig 1:**
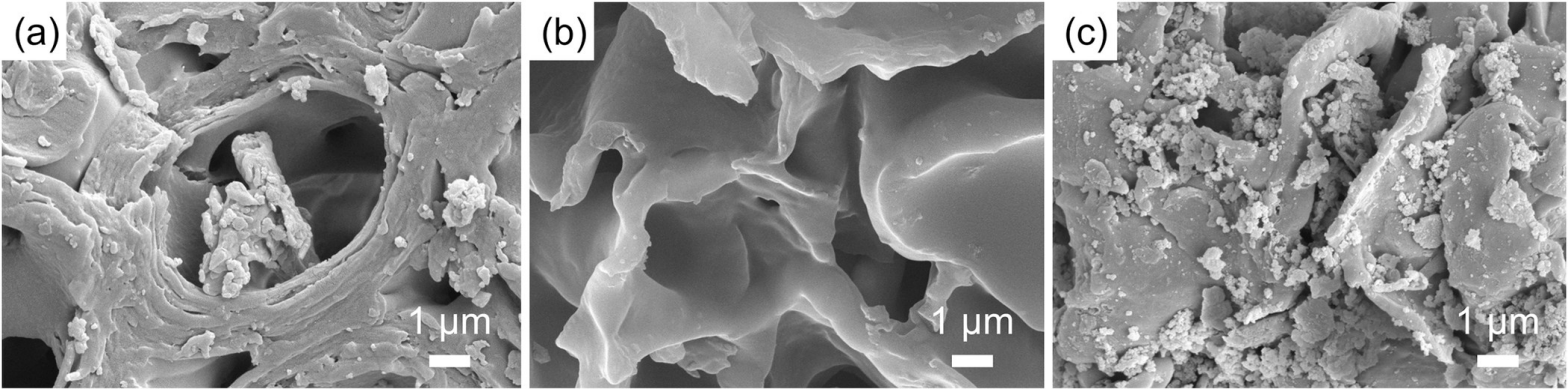
SEM images of raw biomass and biochar: (a) SS; (b) SSB; (c) CaSSB.

The surface morphologies of SS, SSB and CaSSB were assessed by SEM, as shown in Fig 1. The morphology of soybean straw before carbonization in Fig 1A shows wrinkles and some particles on the surface. After pyrolysis, the cellulose, hemicellulose and lignin of straw were destroyed, as they transformed into tar and other sediments, forming evident pores on the SSB (Fig 1B) [36]. As shown in Fig 1C, CaSSB exhibited a rough surface morphology with bulk particles. The forming processes may be described as follows: The calcium ions reacted on the surface of biochar to form particles during modified process, resulting in a rough surface, which increased the adsorption sites of biochar and improved its ion-exchange capacity. The calcium ions were embedded in the carbon structure, causing changes in the crystal structure and pore structure of biochar.

**Fig 2:**
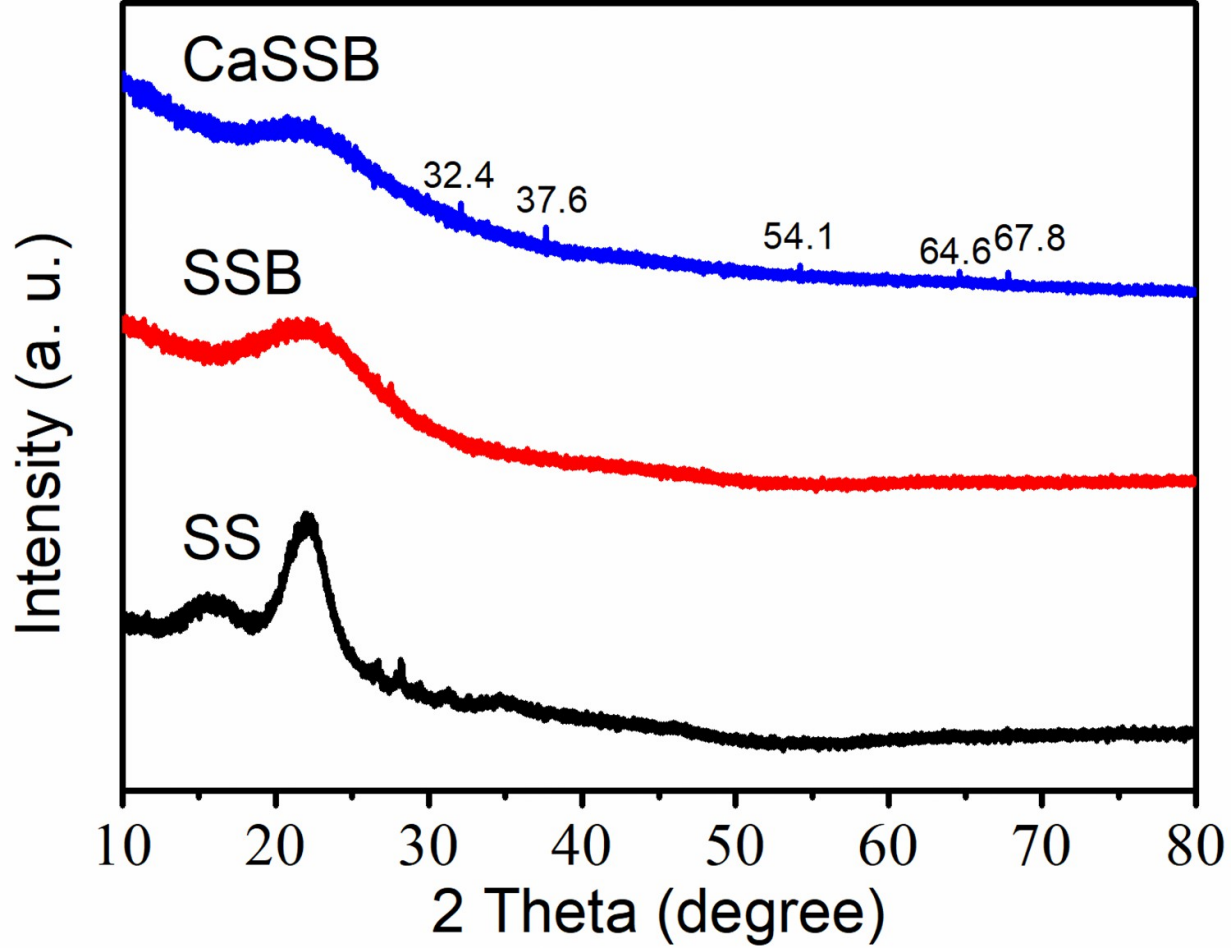
XRD spectra of the SS, SSB and CaSSB.

XRD analysis was used to clarify the crystal structure of SS, SSB and CaSSB. As shown in Fig 2, the XRD spectra of SS, SSB and CaSSB displayed a common diffraction peak at 2θ = 23°, which was indexed as the cellulose or amorphous nature of carbon [37]. The CaSSB contained five distinct wide peaks at 2θ = 32.4°, 37.6°, 54.1°, 64.6° and 67.8°, which putatively corresponded to CaO or Ca(OH)_2_ [38]. These results imply that Ca^2+^ is loaded on the surface of the biochar.

#### FTIR

**Fig 3:**
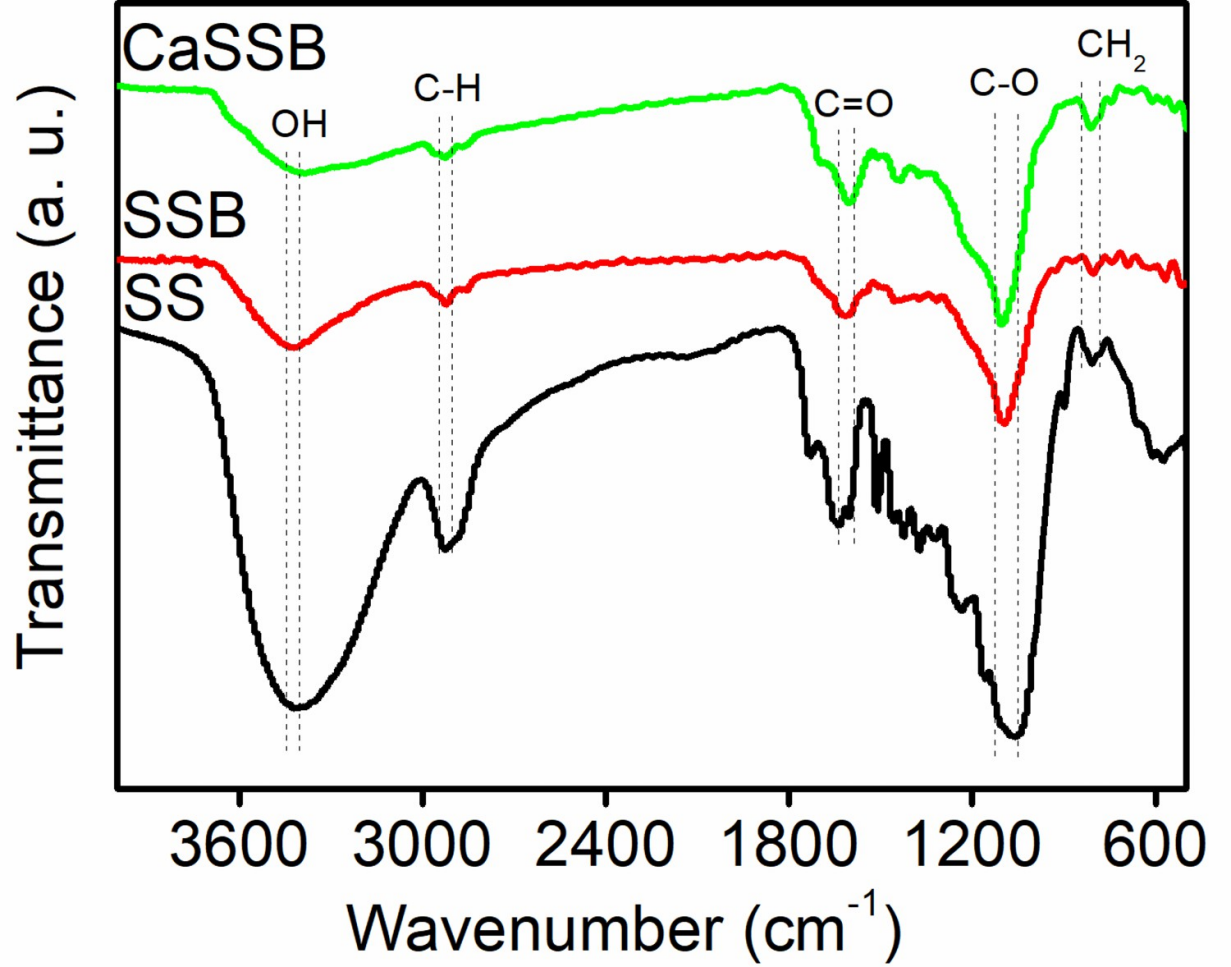
FTIR spectra of raw biomass and biochar.

The FTIR spectra shown in Fig 3 reveal the presence of surface functional groups of samples at wavelengths of approximately 400–4000 cm^−1^. The aromatic, phenolic and aliphatic groups were dominant. The stability of aliphatic carbon compounds was lower than that of aromatic carbon [39]. Therefore, after the carbonization of biomass materials, certain peaks decreased or even disappeared, such as cellulose, carbohydrates, methylene functional groups and so on. According to relevant literature, the absorption peak at 3200–3500 cm^−1^ arose from the O–H stretching vibration of alcoholic or phenolic hydroxyl groups associated with intermolecular hydrogen bonds [40]. The bands in the range of 2856 to 3000 cm^−1^ were produced by the C–H groups of aliphatic saturated. The bands in the range of 1596 to 1620 cm^−1^ represented the aromatic C = O and C = C groups stretching vibration, implying the formation of initial aromatisation of the precursor and carbonyl-containing groups [41]. The bands in the range of 1080–1030 cm^−1^ might be due to the C–O bending vibration in phenolic hydroxy groups. A peak identified at 875–755 cm^−1^ might be attributed to furan CH_2_ groups. The above functional groups in CaSSB also indicated that impregnating CaCl_2_ did not affect the types and structures of the original organic functional groups in soybean biochar [42]. In addition, the bands below 800 cm^−1^ were stronger after CaCl_2_ modification, implying that Ca^2+^ was successfully grafted onto the surface of soybean straw biochar.

### Specific surface area and pore structure

The pore structure and specific surface area of biochar are important factors for the facilitation of ammonia nitrogen and phosphate adsorption in the removal of ammonia nitrogen and phosphate from biogas slurry through biochar [43]. The pore structure characteristics of SS, SSB and CaSSB are listed in Table 3. The specific surface areas of SS, SSB and CaSSB were 1.03, 2.12 and 3.69 m^2^/g, respectively. The average pore diameters varied from 20.89 to 24.87 nm, indicating that most pores were mesopores. The total pore volumes of SS, SSB and CaSSB were 0.0016, 0.0027 and 0.0040 cm^3^/g, respectively. Compared with SS and SSB, CaSSB had a larger specific surface area and lower average pore size owing to the improvement in the pore structure of the biochar by the impregnation of calcium chloride [44].

**Table 3:** Comparisons of surface characteristics of biomass and biochar.

sample	Surface structure			
	Surface area	Average pore diameter	Micropore volume	Total pore volume
	m^2^/g	nm	cm^3^/g	cm^3^/g
SS	1.03	24.87	0.00120	0.0016
SSB	2.12	21.43	0.00051	0.0027
CaSSB	3.69	20.89	0.00068	0.0040

### Ammonia nitrogen and phosphate adsorption

#### Effect of pH

**Fig 4:**
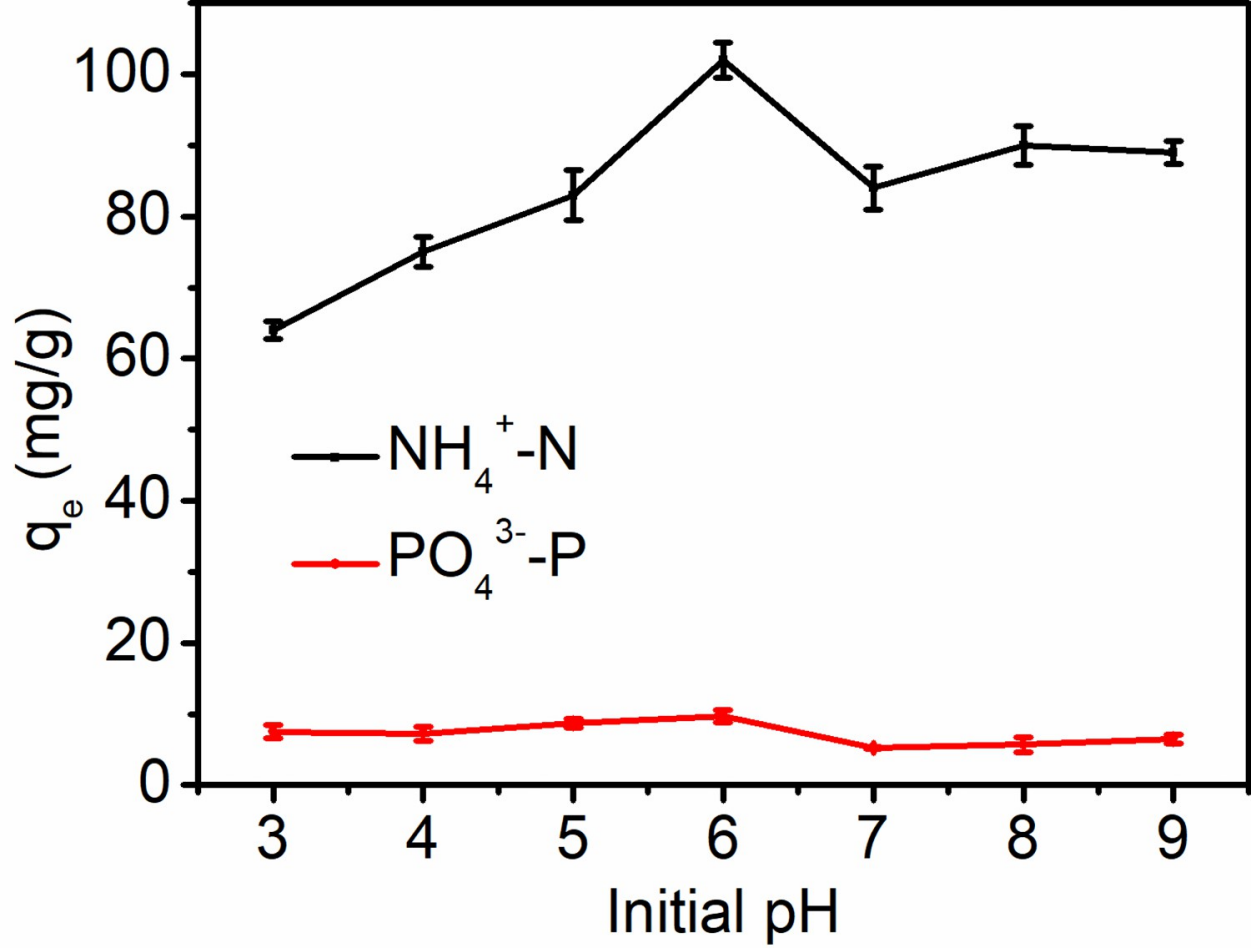
Effect of solution pH_initial_ on adsorption of ammonia and phosphate by CaSSB.

The pH is a key factor for the adsorption performance of biochar, as it directly influences the chemical form of ammonia nitrogen and phosphate, surface charge and the ionization and formation of adsorbates [45]. Fig 4 shows the adsorption performance of ammonia and phosphate of CaSSB at the pH range of 3–10. CaSSB demonstrated the maximum adsorption capacity of ammonia nitrogen (102.21 mg/g) and phosphate (9.75 mg/g) at pH = 6. Strong acidic or alkaline condition would lead to a lower adsorption capacity. These results were similar to those reported by Cheng et al. [14]. Ammonia nitrogen occurs in varying forms of NH_4_^+^–N and NH_3,_ depending on the pH value of the solution. Ammonia nitrogen exists in the form of NH_4_^+^–N when the pH value of the solution is lower than 7, whereas it occurs in the form of NH_4_^+^–N and NH_3_ when the pH is between 7 and 10 [46]. Moreover, the higher the pH value, the higher the free ammonia content, which induces a weakened electrostatic interaction between ammonia and biochar and the inverse effects on the adsorption capacity of biochar [47]. Therefore, the adsorption amount of NH_4_^+^–N by CaSSB increased with the increase in pH from 3–7, and the adsorption exhibited a downward trend at pH > 7.

Phosphate in the solution occurred in the form of H_3_PO_4_, H_2_PO_4_^−^, HPO_4_^2−^, PO_4_^3−^, of which H_2_PO_4_^−^ is most likely to replace OH^−^ and combine with Ca^2+^, followed by HPO_4_^2−^. Similarly, phosphorus exists in different forms, depending on the pH value: PO_4_^3−^ at pH > 12.33; HPO_4_^2−^ at 7.2 < pH < 12.33; H_2_PO_4_^−^ at pH < 7.2 [48]. Therefore, in acidic conditions, the amount of phosphate adsorbed by CaSSB increased with the increase in pH. Meanwhile, in alkaline conditions, the opposite is true. These results were similar to Wang [49] and Blaney [50].

#### Effect of dosage

**Fig 5:**
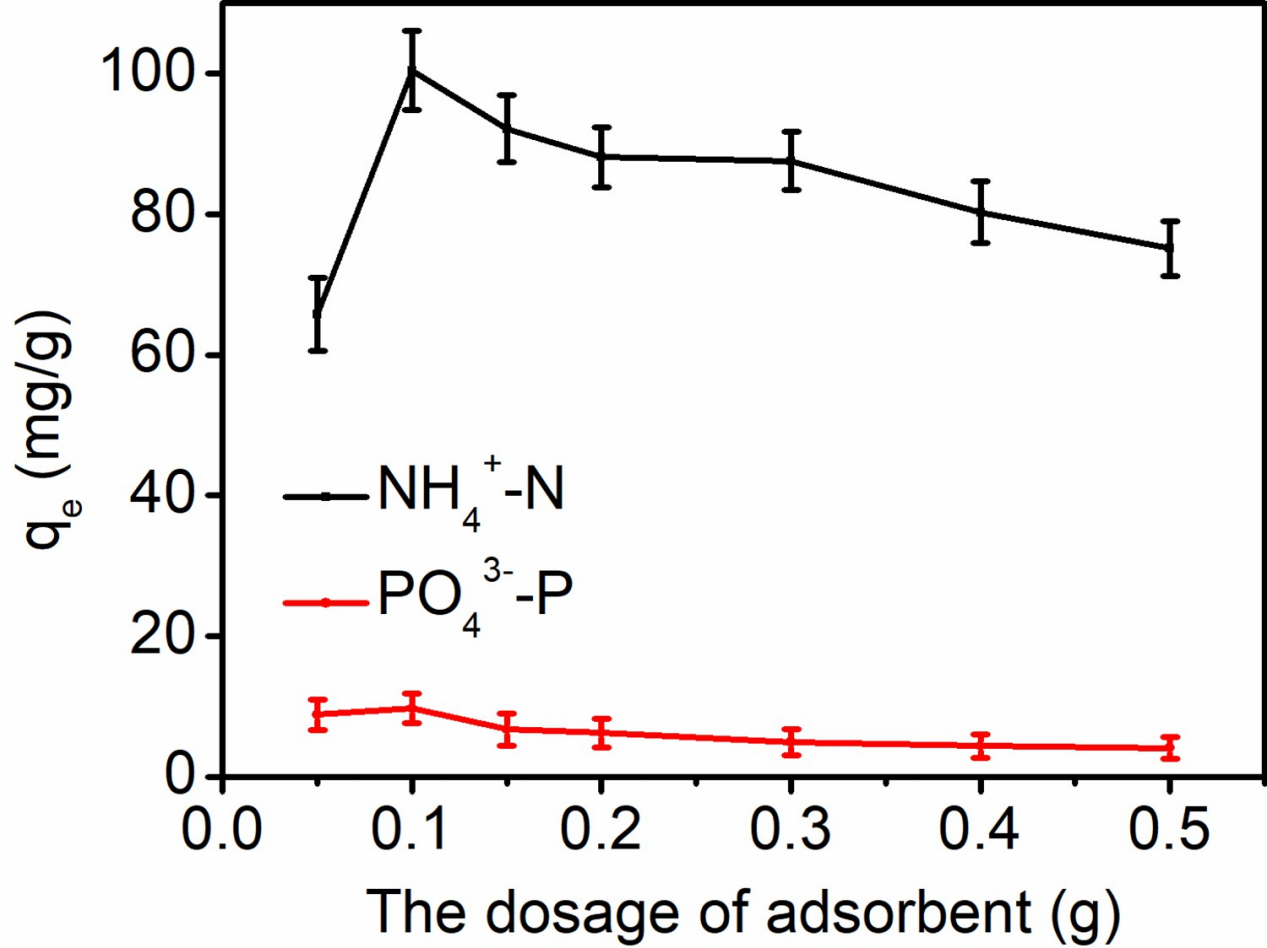
Adsorption performance of CaSSB for ammonia nitrogen and phosphate under different dosages.

The impact of adsorbent dosage on the adsorption capacity of ammonia nitrogen and phosphate by CaSSB was investigated, and the results are shown in Fig 5. The adsorption capacity of ammonia and phosphate increased with the dosage of CaSSB. Specifically, the adsorption capacity of ammonia nitrogen increased from 65.73 to 100.43 mg/g with the increase in CaSSB dosage from 0.05 to 0.1 g. Similarly, the adsorption capacity of phosphate increased from 8.83 to 9.71 mg/g when the CaSSB dosage increased from 0.05 to 0.1 g. Nonetheless, when the CaSSB dosage was increased from 0.1 to 0.5 g, a slight reduction could be observed in the adsorption capacity of ammonia nitrogen and phosphate. The enhanced removal efficiency at lower adsorbent dosages might be attributed to an increase in the available active sorption sites on CaSSB. The decline in the unit adsorption capacity of ammonia nitrogen and phosphate was observed when the dosage exceeded 0.1 g. This phenomenon can be attributed to the fact that higher adsorbent dosages may lead to a reduction in the unsaturation of the ion-exchange sites on CaSSB, resulting in a decline in the amount of ion-exchange sites per unit mass. Considering the nutrient adsorption capacity, efficiency and economic factors, the optimal dosage for the CaSSB was determined to be 2 g/L.

### Effect of coexisting anions

**Fig 6:**
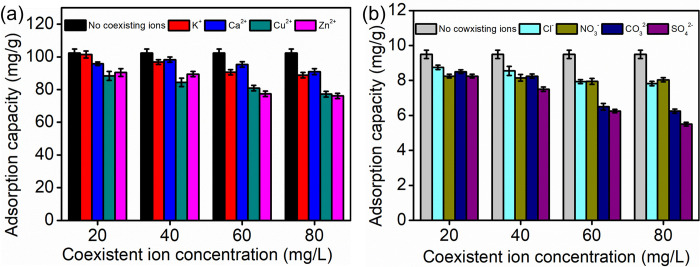
Coexisting ions effect on CaSSB adsorption performance: (a) ammonia nitrogen and (b) phosphate.

Biogas slurry usually contains metal ions and inorganic salts, and these ions may engage in competition with ammonia nitrogen and phosphate for the adsorption sites of CaSSB. As shown in Fig 6A, different ions exhibited varying degrees of effects on the adsorption performance of CaSSB. When the concentration of K^+^ and Ca^2+^ was lower than 40 mg/L, the ion concentrations had a negligible impact on the adsorption performance of CaSSB for the ammonia nitrogen. Cu^2+^ and Zn^2+^ exerted a greater influence on the adsorption process with the increase in concentration. This phenomenon is similar to the results obtained by Liu et al. [51]; it may be attributed to the competition between the metal cation with ammonium ions for the adsorption sites. For phosphate adsorption, Yao et al. [52] determined that when using a synthetic adsorption solution containing phosphate, Cl^−^, NO_3_^−^, HCO_3_^−^ and humic acid—similar to actual wastewater—the biochar’s phosphate adsorption capacity decreased by 40% compared to that in a pure phosphate solution. However, Cl^−^, CO_3_^2−^, SO_4_^2−^ and NO_3_^−^ have a negligible impact on the phosphate adsorption by CaSSB when their concentrations are below 40 mg/L. On the contrary, the adsorption capacity of CaSSB for phosphate decreased when the concentration of coexisting anions increased. Among them, CO_3_^2−^ and SO_4_^2−^ exerted a greater negative impact. It is speculated that they can react with calcium to produce precipitation, which reduces the adsorption sites for phosphate.

### Comparison of adsorption capacities of SSB and CaSSB

**Fig 7:**
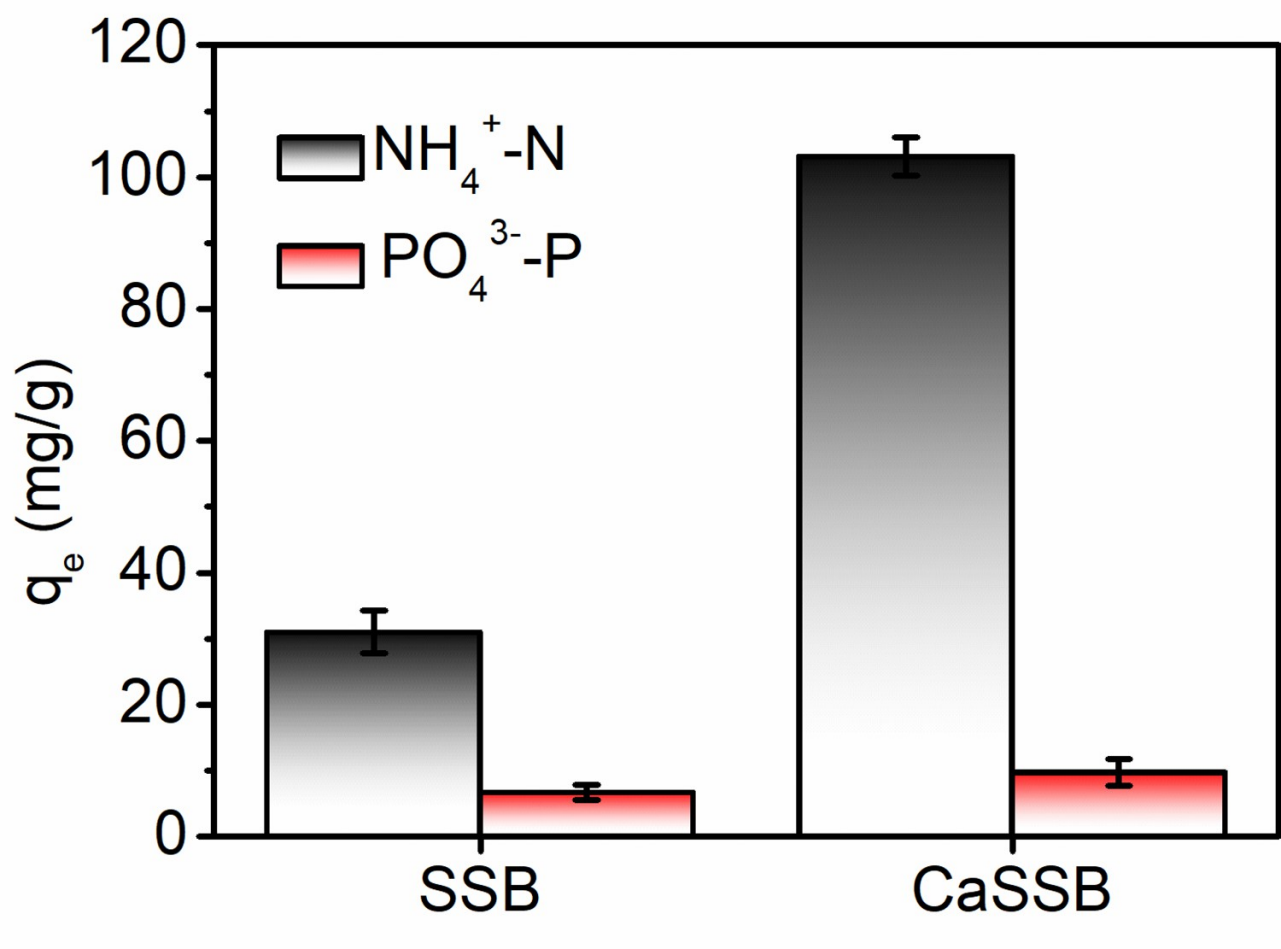
Comparison of adsorption capacities of SSB and CaSSB for ammonia nitrogen and phosphate in biogas slurry.

The maximum adsorption performances for ammonia nitrogen and phosphate obtained by CaSSB and SSB under optimal conditions are shown in Fig 7. The adsorption capacities of CaSSB for ammonia nitrogen and phosphate were 103.18 and 9.75 mg/g, respectively, remarkably higher values than those of SSB (31.0 and 6.69 mg/g, respectively). The maximum adsorption capacities of the CaSSB for ammonia nitrogen and phosphate were 3.33 and 1.45 times those of SSB. These results revealed that the adsorption performance of SSB for ammonia and phosphate in biogas slurry considerably improved after modification with the calcium ion. The adsorption capacity of CaSSB to ammonia nitrogen was remarkably greater than that of the phosphate. The adsorption capacity of ammonium increased as a result of the negative charge of biochar. Overall, negatively charged biochar underperformed in removing anionic pollutants. The high calcium content of CaSSB was responsible for its efficient removal of phosphate, as it could form metal bonds, such as Ca–O–P, through precipitation with phosphate acid. The adsorption effects of CaSSB and other adsorbents reported previously on ammonia and phosphate are listed in Table 4. Herein, the soybean straw–based biochar was modified with Ca^2+^, which exhibited high adsorption capacity for ammonia nitrogen and phosphate in comparison to most previously reported adsorbents. This result also suggested CaSSB could be used to recover ammonium and phosphate from biogas slurry.

**Table 4:** Maximum ammonia nitrogen and phosphate adsorption capacity of CaSSB and other adsorbents.

Adsorbent	NH_4_^+^–N	PO_4_^3−^	Dose	Reference
	(mg/g)	(mg/g)	(g/L)	
CO_2_-modified *Phragmites australis* biochar	4.0	<0	4	Zeng et al. 2013 [53]
CO_2_-modified Thalia ealbata	17.6	3.0	4	Zeng et al. 2013 [53]
MgCl_2_-modified Peanut shells	/	6.3	2	Zhang et al. 2012 [54]
MgCl_2_-modified Chinese fir straw biochar	18.48	6.77	5	He et al. 2022 [24]
Hardwood shavings	5.3	0.2	2	Sarkhot et al. 2013 [55]
oil‐palm‐shell biochar	<1.49	/	6	Munar–Florez, et al. 2021 [56]
oil‐palm‐shell biochar	/	<0.89	14	Munar–Florez, et al. 2021 [56]
NaCl modified zeolite	11.25	6.67	30	Cheng et al. 2017 [14]
*Eupatorium adenophorum* biochar	1.91	2.32	2.5	Cheng et al. 2021 [34]
Ca^2+^-modified soybean straw biochar	103.18	9.75	2	This work

### Adsorption kinetics

**Fig 8:**
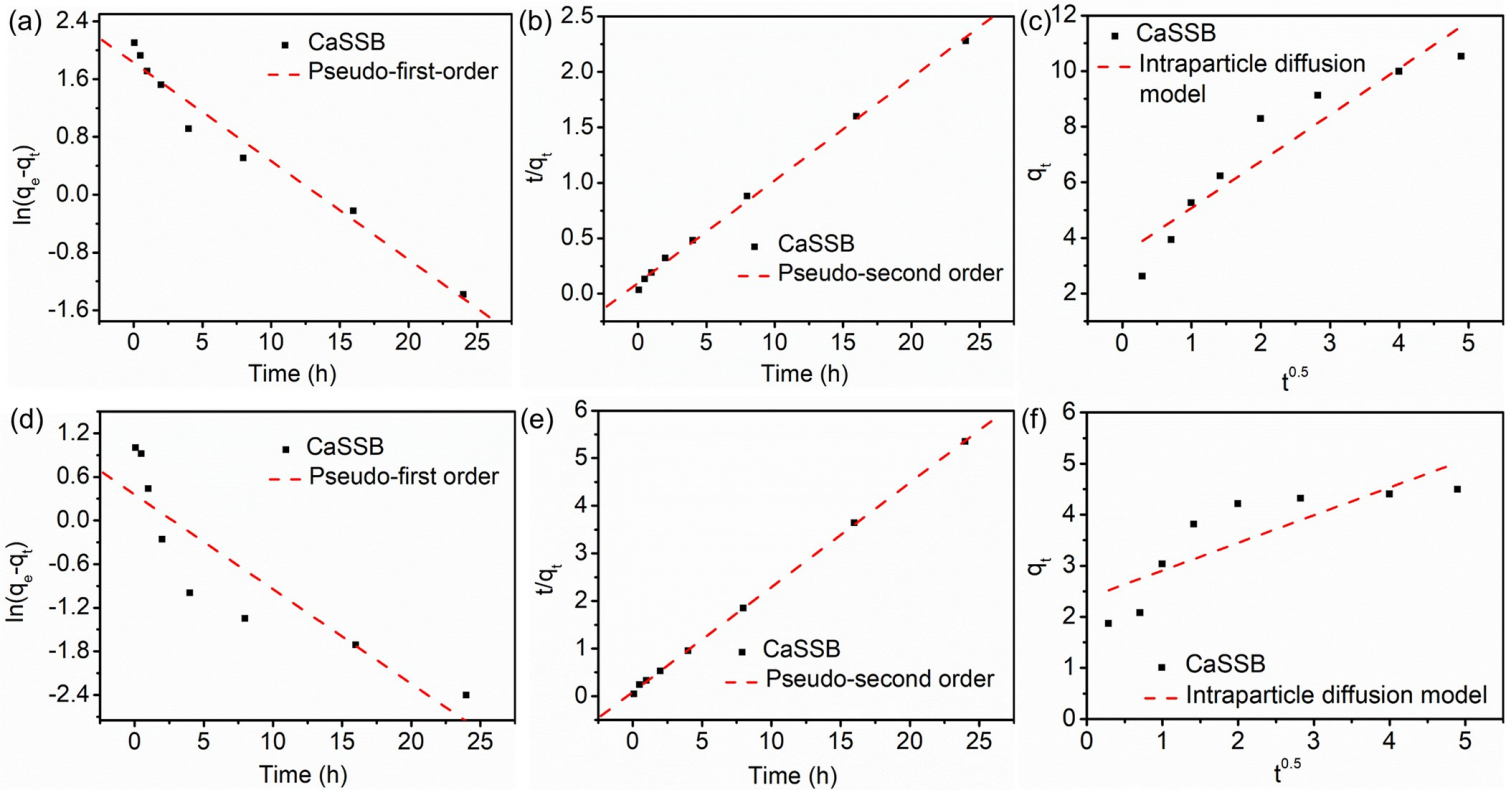
Adsorption kinetics of ammonia nitrogen (a-c) and phosphate (d-f) on CaSSB: following pseudo-first-order kinetics for ammonia nitrogen (a) and phosphate (d); pseudo-second-order kinetics for ammonia nitrogen (b) and phosphate (e); intraparticle diffusion model for ammonia nitrogen (c) and phosphate (f).

The adsorption kinetics of ammonia and phosphate on CaSSB were studied. The initial concentrations of ammonia nitrogen and phosphate were 30 and 20 mg/L, respectively. The initial pH of the solutions for the adsorption kinetics for both experiments was 6.0. The adsorption processes of CaSSB for ammonia and phosphate were recorded during 24 h tests. Fig 8 presents the ammonia and phosphate adsorption kinetics of CaSSB. The adsorption process of CaSSB for ammonia nitrogen followed a typical kinetic model, with a rapid sorption rate in the first 30 minutes, followed by an increase in the adsorption capacity as time progressed. The adsorption equilibrium time was found to be 24 hours, and therefore, all batch experiments were shaken for 24 h. To explore the reaction mechanisms, the pseudo-first-order, pseudo-second-order model and the intraparticle diffusion model were used to fit the experimental data. The corresponding kinetic parameters, k_1_, k_2_, k_3_ and a, calculated q_e_ values and the correlation coefficients R^2^, were shown in Table 5. The results suggested that q_e_ values of ammonia nitrogen and phosphate calculated using the pseudo-second order kinetics were more aligned with the experimental values than those of other models. The q_e_ values of ammonia nitrogen and phosphate calculated using the pseudo-second order kinetics were 10.811 and 4.547 mg/g, respectively. Compared to the pseudo-first order and intraparticle diffusion model, the correlation coefficients of the pseudo-second order kinetic equation was higher, suggesting its suitability for the entire adsorption process. These results also indicated that the adsorption of ammonia nitrogen and phosphate process might be dominated by chemical reactions, such as cation exchange, sedimentation and complexation. The maximum removal rates of CaSSB for ammonia nitrogen and phosphate were 71.87% and 45.80%, respectively. The saturated adsorption amount of CaSSB surpassed that of reported biochar materials derived from phytoremediation plants, cornstalk, wood chip and other sources [21, 53].

**Table 5:** Adsorption kinetic parameters for ammonia nitrogen and phosphate on CaSSB.

Sample	qe,exp (mg/g)	Pseudo-first order			Pseudo-second order			Intraparticle diffusion		
		k_1_ (min^−1^)	q_e,cal_ (mg/g)	R^2^	k_2_ (min^−1^)	q_e,cal_ (mg/g)	R^2^	k_3_ (mmol kg^−1^ h^−0.5^)	a	R^2^
NH_4_^+^–N	10.780	0.136	6.223	0.969	0.0887	10.811	0.998	1.675	3.405	0.868
PO_4_^3−^–P	4.580	0.132	1.430	0.800	0.553	4.547	0.999	0.542	2.365	0.647

### Adsorption isotherm

**Fig 9:**
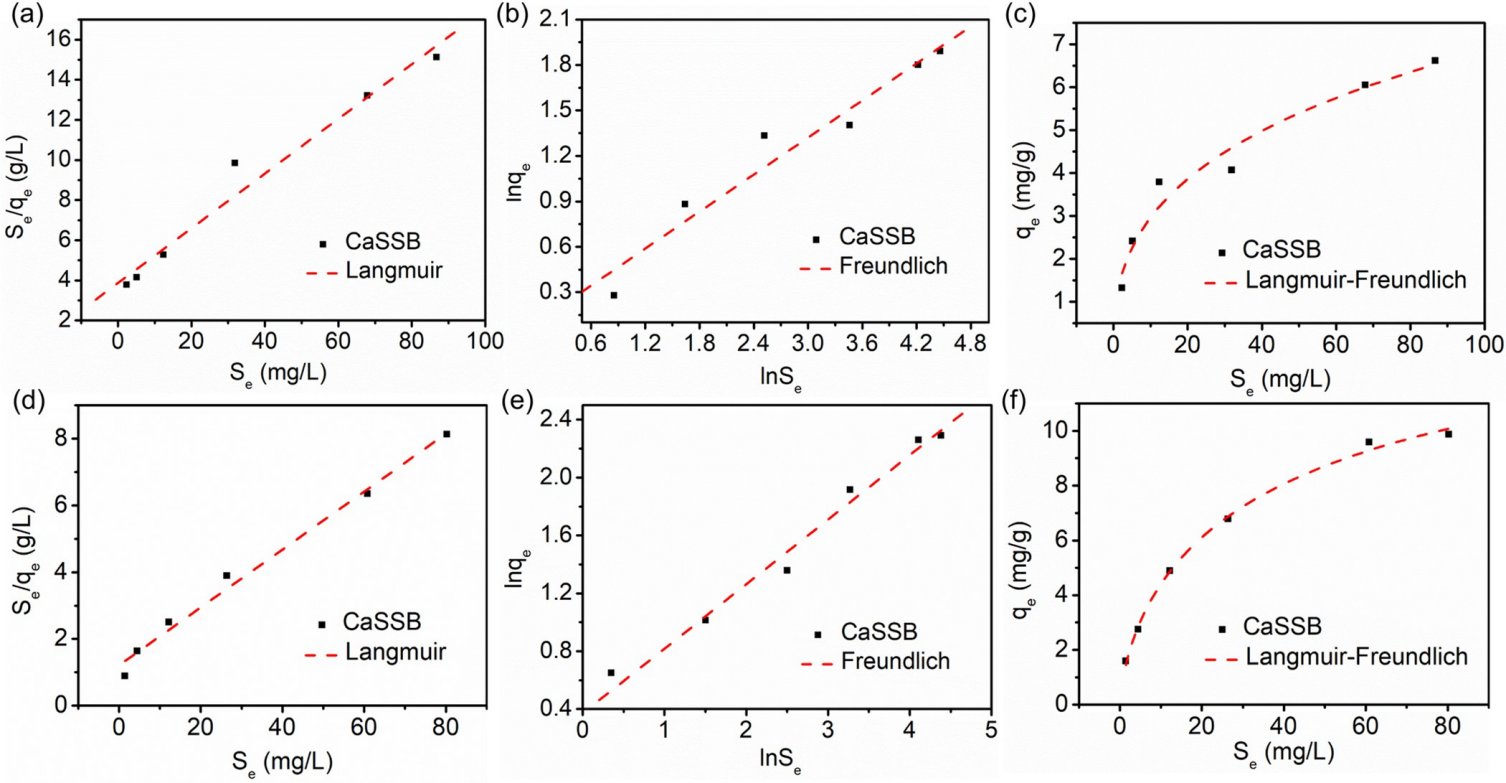
Adsorption isotherms of ammonia nitrogen (a-c) and phosphate (d-f): following the Langmuir model for ammonia nitrogen (a) and phosphate (d); the Freundlich model for ammonia nitrogen (b) and phosphate (e); the Langmuir–Freundlich for ammonia nitrogen (c) and phosphate (f).

As shown in Fig 9, the adsorption isotherms of CaSSB for ammonia and phosphate were tested with various initial concentrations in the range of 0–100 mg/L. The experimental data was modelled with Langmuir, Freundlich and Langmuir–Freundlich models. Table 6 presents the adsorption parameters and correlation coefficients for ammonia nitrogen and phosphate on CaSBB obtained from the three models. The correlation coefficient for the Langmuir model (R^2^: 0.972 for NH_4_^+^–N) was slightly higher compared to those for the Freundlich (R^2^: 0.947 for NH_4_^+^–N) and Langmuir–Freundlich models (R^2^: 0.941 for NH_4_^+^–N), indicating a better fit for the Langmuir model. The Langmuir model may imply monolayer adsorption for ammonia nitrogen on the surface of homogeneous adsorbents [57]. The surface of CaSBB exhibited a potentially finite number of uniform adsorption sites with consistent ammonia adsorption affinities [58]. Hence, it is likely that the mechanism involved in the ammonia adsorption process was monolayer adsorption, primarily occurring on the surface-active regions of the CaSBB. However, among the three models, the Langmuir–Freundlich model provided the highest fitting accuracy (R^2^: 0.995 for PO_4_^3−^–P) for the phosphate on CaSBB. It is deduced that the adsorption of phosphate on CaSSB is a physicochemical adsorption process. The dominant sorption mechanism of CaSSB for phosphate might be the reaction between Ca^2+^ and OH^−^ with PO_4_^3−^ to generate Ca_5_(PO_4_)_3_(OH) precipitation. The parameter K_L_, which represents the affinity of the binding sites, enables a comparison of the biochar’s affinity towards ammonia and phosphate. The affinity of CaSBB for ammonia nitrogen (1.895 L/mg) was higher than that of phosphate (0.071 L/mg). It can be inferred from the obtained 1/n values of ammonia nitrogen and phosphate being <0.5, that CaSBB demonstrates superior adsorption performance for ammonia nitrogen and phosphate.

**Table 6:** Adsorption isotherm parameters for ammonia nitrogen and phosphate sorption by CaSBB.

Sample	Langmuir model			Freundlich model			Langmuir–Freundlich			
	K_L_ (L/mg)	q_max (_mg/g_)_	R^2^	K_F_ (mg/g)	1/n	R^2^	q_max (_mg/g_)_	K_LF_ (L/mg)	n	R^2^
NH_4_^+^–N	1.895	7.331	0.972	1.104	0.407	0.947	30.61	0.039	0.430	0.941
PO_4_^3−^–P	0.0710	11.530	0.990	1.542	0.416	0.982	17.262	0.072	0.677	0.995

## Conclusions

The Ca^2+^-modified soybean straw biochar was prepared and used for adsorbing ammonia nitrogen and phosphate from biogas slurry. The zeta potential, BET surface area, elemental analysis and the adsorption capacity of the CaSSB were analysed and investigated. After modification with CaCl_2_, the CaSSB derived from soybean straw exhibited a considerable adsorption capacity for ammonia nitrogen (103.18 mg/g) and phosphate (9.75 mg/g), demonstrating the feasibility of the proposed method for treating biogas slurry. In addition, the adsorption kinetics of ammonia and phosphate on CaSSB were examined using three kinetic models. The experimental results of CaSSB on ammonia nitrogen and phosphate conform to the pseudo-second-order kinetic model, indicating that chemical reactions might have taken place during the adsorption process. Furthermore, the maximum removal rates of ammonia nitrogen and phosphate by CaSBB were found to be 71.87% and 45.80%, respectively. In comparison with the Freundlich isotherm model, the Langmuir model was found to be more appropriate for characterising the adsorption of ammonia nitrogen on CaSSB, whereas the Langmuir–Freundlich model was more suitable for the adsorption of phosphate. These results suggest that the adsorption might be attributed to monolayer adsorption and involved the chemical process. The adsorption mechanisms of CaSSB could be Ca–P precipitation, ion exchange and electrostatic attraction. The results indicate that the modification of biochar by calcium chloride improves its adsorption capacity for biogas slurry nutrients, and that CaSBB is a promising material for biogas slurry resource treatment.

## Supporting information

S1 File Effect of coexisting anions and comparison of the adsorption capacity of the SSB and CaSSB. (DOC)
