# Supplementary material for: Adsorption characteristics and mechanism of ammonia nitrogen and phosphate from biogas slurry by Ca2+-modified soybean straw biochar
Source: PLoS One. 2023 Aug 25;18(8):e0290714. doi: 10.1371/journal.pone.0290714 (PMC10456179; doi:10.1371/journal.pone.0290714)
Supplement: S1 File — (DOC) [file pone.0290714.s001.doc]

Supporting Information

***Adsorption characteristics and mechanism of*** ***ammonia nitrogen and phosphate from biogas slurry by*** ***Ca^2+^-modified soybean straw biochar***

Xiaomei Wu^1,2^*, Meifeng Ye^1^, Jinglong Wang^3^, Feilong Wu^1^, Cenwei Liu^4^, Zhangting Li^1^, Daiyan Lin^1^, Rilong Yang^2^*

minimal data set

1.**Effect of coexisting anions**

The various concentrations of K^+^, Ca^2+^, Cu^2+^, Zn^2+^, Cl^-^, CO_3_^2-^, SO_4_^2-^ and NO_3_^-^ were respectively added into the 474.00 mg/L ammonium and 50.00 mg/L phosphate solution before the addition of 2 g/L CaSSB.

S1 Table Effect of coexisting ions on the adsorption of ammonium nitrogen by CaSSB

| Coexistent ion concentration  mg/L | no coexisting ions | K^+^ | Ca^2+^ | Cu^2+^ | Zn^2+^ |
| --- | --- | --- | --- | --- | --- |
|  | mg/g | | | | |
| 20 | 102.425±2.341 | 101.425±2.131 | 95.825±1.130 | 88.325±2.722 | 90.425±2.401 |
| 40 | 102.425±2.341 | 96.825±1.442 | 98.325±1.623 | 84.425±2.632 | 89.425±1.651 |
| 60 | 102.425±2.341 | 90.575±1.672 | 95.325±1.742 | 80.925±1.753 | 77.425±1.711 |
| 80 | 102.425±2.341 | 88.825±1.723 | 90.925±1.820 | 77.175±1.711 | 76.145±1.642 |

S2 Table Effect of coexisting ions on the adsorption of phosphate by CaSSB

| Coexistent ion concentration  mg/L | no coexisting ions | Cl^-^ | NO_3_^-^ | CO_3_^2-^ | SO_4_^2-^ |
| --- | --- | --- | --- | --- | --- |
|  | mg/g | | | | |
| 20 | 9.512±0.243 | 8.751±0.127 | 8.255±0.107 | 8.504±0.106 | 8.254±0.106 |
| 40 | 9.512±0.243 | 8.552±0.256 | 8.151±0.191 | 8.250±0.12 | 7.504±0.124 |
| 60 | 9.512±0.243 | 7.931±0.112 | 7.950±0.165 | 6.512±0.19 | 6.251±0.104 |
| 80 | 9.512±0.243 | 7.822±0.127 | 8.044±0.114 | 6.250±0.12 | 5.503±0.108 |

2.Comparison of the adsorption capacity of the SSB and CaSSB

To assess the applicability of the CaSSB adsorbent for actual wastewater, 0.1g CaSSB was added to the 50 mL biogas slurry. The test container is a 250 mL glass Erlenmeyer flask, and the volume of each test adsorption solution is 50 mL. Adsorption process were carried out at room temperature with shaking at a speed of 150 r/min for 24 h. When the adsorption was complete, the sample to be tested was filtered by a 0.45 µm filter for the measurement of ammonia nitrogen and phosphate.

S3 Table Comparison of the adsorption capacity of the SSB and CaSSB (mg/g)

| Sample | The adsorption capacity | |
| --- | --- | --- |
|  | ammonia nitrogen | phosphate |
| SSB | 103.180±2.891 | 9.750±2.030 |
| CaSSB | 31.000±3.242 | 6.690±1.094 |
